# Assessing Tuberculosis Case Fatality Ratio: A Meta-Analysis

**DOI:** 10.1371/journal.pone.0020755

**Published:** 2011-06-27

**Authors:** Masja Straetemans, Philippe Glaziou, Ana L. Bierrenbach, Charalambos Sismanidis, Marieke J. van der Werf

**Affiliations:** 1 Unit Knowledge, Research and Policy, KNCV Tuberculosis Foundation, The Hague, The Netherlands; 2 Department of Clinical Epidemiology, Biostatistics and Bioinformatics, Center for Infection and Immunity Amsterdam (CINIMA), Academic Medical Center, University of Amsterdam, Amsterdam, The Netherlands; 3 Stop TB Department, World Health Organization, Geneva, Switzerland; McGill University, Canada

## Abstract

**Background:**

Recently, the tuberculosis (TB) Task Force Impact Measurement acknowledged the need to review the assumptions underlying the TB mortality estimates published annually by the World Health Organization (WHO). TB mortality is indirectly measured by multiplying estimated TB incidence with estimated case fatality ratio (CFR). We conducted a meta-analysis to estimate the TB case fatality ratio in TB patients having initiated TB treatment.

**Methods:**

We searched for eligible studies in the PubMed and Embase databases through March 4^th^ 2011 and by reference listing of relevant review articles. Main analyses included the estimation of the pooled percentages of: a) TB patients dying *due to* TB after having initiated TB treatment and b) TB patients dying *during* TB treatment. Pooled percentages were estimated using random effects regression models on the combined patient population from all studies.

**Main Results:**

We identified 69 relevant studies of which 22 provided data on mortality *due to* TB and 59 provided data on mortality *during* TB treatment. Among HIV infected persons the pooled percentage of TB patients dying *due to* TB was 9.2% (95% Confidence Interval (CI): 3.7%–14.7%) and among HIV uninfected persons 3.0% (95% CI: −1.2%–7.4%) based on the results of eight and three studies respectively providing data for this analyses. The pooled percentage of TB patients dying *during* TB treatment was 18.8% (95% CI: 14.8%–22.8%) among HIV infected patients and 3.5% (95% CI: 2.0%–4.92%) among HIV uninfected patients based on the results of 27 and 19 studies respectively.

**Conclusion:**

The results of the literature review are useful in generating prior distributions of CFR in countries with vital registration systems and have contributed towards revised estimates of TB mortality This literature review did not provide us with all data needed for a valid estimation of TB CFR in TB patients initiating TB treatment.

## Introduction

Each year, the World Health Organization (WHO) publishes country-specific estimates of tuberculosis (TB) incidence, TB prevalence and TB mortality [1]. In countries without adequate data from national vital registration systems, TB deaths are indirectly estimated by multiplying estimated TB incidence with an estimate of the case-fatality ratio (CFR), accounting for uncertainty in incidence and CFR [2]. Such indirect TB mortality estimates heavily depend on the reliability of underlying estimates of incidence and CFR [3]. The CFR is defined as the probability of dying from a disease before recovering or dying of something else [4]. The TB CFR is defined as the proportion of TB patients dying *due to* TB [5]. In the past, the TB CFR estimates used by WHO and reported in several publications and journals [3], [5]–[7] were based on literature searches assessing mortality during TB treatment; risk of TB relapse and late complications; autopsy series for the cause of death in patients with TB according to human immunodeficiency virus (HIV) status, smear status and treatment regimen. The results from the literature searches, along with treatment results reported to the WHO and country-specific estimates calculated for 1997, were used to estimate CFRs for patients with TB. The resulting CFR for TB patients varied between countries [5]. Other non systematic literature reviews have also reported CFRs in TB patients [8], [9]. Mukadi *et al.* [8] provided a limited description of the review methodology, however, it remained unclear how results of individual studies contributed to the selection of CFR values for TB mortality estimation purposes. Maher *et al.* defined TB CFR as the proportion of TB patients that died within a specified time, without any specification of cause of death [9].

In 2008 it was acknowledged by the Task Force Impact Measurement that the TB CFR needed to be reviewed by a combination of methodologies that included assessment of available data; comparison of notification data and vital registration data in countries that have ‘reliable’ data from these two sources and using data from the literature [10]. To meet this need we have conducted a systematic literature review including a meta-analysis to estimate the TB CFR in TB patients initiating TB treatment, by identifying published studies in which information on TB mortality in a cohort of TB patients receiving TB treatment is available. By summarizing the results obtained from the identified studies we aim to contribute to a revision of the CFR for TB patients initiating TB treatment currently being used by WHO when estimating TB mortality [6], [11]. The methodology and results of the estimates for the duration and CFR in untreated TB patients are reported elsewhere [12].

## Methods

A protocol was developed in advance of conducting the systematic review.

### Search strategy

To identify studies reporting TB CFR in TB patients initiating TB treatment or information that can be used to calculate the CFR, we searched the PubMed and Embase databases through March 4^th^ 2011. We combined the search strategy of the free text terms ‘case fatality’ AND ‘tuberculosis’ with the ‘OR’ operator including the exploded MESH headings ‘mortality’ AND ‘tuberculosis’ AND cause of death’. The search was limited to humans, and we excluded from the search strategy the publication type ‘letter’. We further performed reference listing of relevant review articles that were identified in the search [5], [8], [11], [13], [14] and review articles not identified in the search but which were known by the authors [3], [9], [15].

### Eligibility criteria and study selection

Eligible studies included cohort studies, clinical trials and case control studies. Case control studies were only included if the manuscript reported data on mortality for the group of patients starting TB treatment in addition to conducting the case control analyses. The population studied included pulmonary and extrapulmonary TB patients of both sexes and all ages who initiated TB treatment. The studies needed to report on TB treatment and mortality *due to* TB or mortality *during* TB treatment. We excluded studies conducted in limited populations as they were less likely to be representative of the average TB patient, for example studies of patients with liver cirrhosis [16], health care workers [17], [18] or patients in intensive care units [19]. Furthermore, we excluded non-English articles and, in an attempt to minimize bias, we also decided a posteriori to exclude one article in which authors stated that their recorded mortality data were clearly inaccurate [20]. Identified studies were reviewed for eligibility by one author (MS) based on title and abstract. The full text article was obtained for those articles of which the abstract reported data on case fatality ratio, prospective or retrospective studies in a TB cohort with report of mortality data, cohort study with measurement of incident TB and mortality. For reference listing of the eight review articles [3], [5], [8], [9], [11], [13]–[15] we decided to retrieve the full text of the article if the citation in the review article reported CFR or if the title was indicative of measurement of mortality in treated TB patients.

### Data extraction

One reviewer (MS) extracted data from all eligible studies and a second reviewer (MvdW) independently extracted data from a random subset of 20% of the articles. No major discrepancies were identified in the most important variables. Data extraction included: information on first author; year of publication; study design; country in which the study was performed; setting; coverage; study period; age of cohort initiating TB treatment, type of TB patient (e.g. new, re-treated); TB form (pulmonary, extra pulmonary); type of treatment (standardized versus non-standardized regimen) and duration, Human Immunodeficiency Virus (HIV)- status; multi drug resistance (MDR) status; smear status; whether TB had been registered as cause of death or whether mortality during TB treatment was reported; number of TB patients receiving TB treatment and number of TB patients died.

Although we originally planned to extract data on follow up time, number of patients lost to follow up, use of cotrimoxazole prophylaxis, co-morbidities and use of anti-retroviral therapy, these data appeared to be limited and consequently could only be partly considered in the description of the retrieved studies.

We classified TB treatment as standardized if 1) the described treatment regimen was in line with WHO recommendations as described in guidelines published in 1997 [21] or 2003 [22]; 2) the treatment regimen was described as following the national/governmental guidelines; 3) the treatment regimen was described as direct observed treatment strategy (DOTS); 4) the treatment regimen was described as ‘standard’. We classified TB treatment as non-standardized if 1) TB treatment was described as ‘individual tailored regimen’; 2) the treatment regimen was described as ‘according to non-governmental organization’ (NGO); 3) the treatment regimen was described as ‘unsupervised ambulatory’. If the treatment regimen was described as unknown, this was categorized as unknown treatment regimen. These studies were considered as having received non-standardized TB treatment. In two studies part of the cohort received standard TB treatment and part non standard [23], [24]. Although we originally planned to present the results differently for those patients receiving standardized and non-standardized treatment the numbers were too small. To assess the robustness of our results we conducted a sensitivity analyses by excluding studies in which treatment was categorized as non-standardized.

### Operationalisation of outcome measures

The primary outcome measure was the percentage of TB patients initiating TB treatment that died *due to* TB within a specific time period. This was defined as TB CFR and was calculated by dividing the number of TB patients dying *due to* TB by the total number of TB patients in the cohort initiating TB treatment, multiplied by 100. Studies having reported the registration of cause of death and having reported TB as either ‘primary cause of death’, ‘contributory cause of death’, ‘immediate cause of death’ or ‘related to TB’ were included in this analysis. The secondary outcome measure was the percentage of TB patients dying *during* TB treatment, irrespective of cause of death.

### Quality assessment

No quality assessment of studies was done. Existing quality assessment tools were judged not to be relevant for the quality assessment of the type of studies included and the outcome measure of interest. Furthermore, it was expected that the use of a quality assessment tool would not result in a large variability in quality assessment scores of the individual studies.

### Synthesis of results

For our main analyses we have calculated the pooled percentage of deaths *due to* TB (primary outcome measure) and the pooled percentage of overall deaths *during* TB treatment (secondary outcome measure) based on the individual studies. To estimate the pooled percentages of death across all studies, accounting for the clustering effect (at the level of the study), and the standard error (s.e.) of the point prevalence, we used random effect regression models. Models were fitted using individual patients as the unit of analysis, achieved after appropriate expansion and pooling of the aggregated counts of patients who died and survived in each study. We present the estimated pooled prevalence with the 95% confidence interval (CI).

Originally we planned to conduct subgroup analyses for new and retreated TB patients. However, the studies did not always make this distinction and/or the number of studies in each category was limited. Therefore, we only present data for new and re-treated TB patients combined. We also planned to conduct separate analyses for different age categories, but the studies did not provide separate results for different age categegories. Information for the following subgroups: HIV status, smear status; multi-drug-resistance (MDR) TB and combinations within these categories are presented when available.

For our secondary analyses we included studies providing information on both the total number of TB patients dying *during* TB treatment and the number of TB patients dying *during* TB treatment *due* to TB, to estimate the pooled percentage of total deaths during TB treatment that were attributed to TB. The pooled percentage of overall mortality within four years *after* TB treatment initiation was also assessed. Analyses were done with the statistical software STATA 11.0.

## Results

### Identified studies

**Figure 1:**
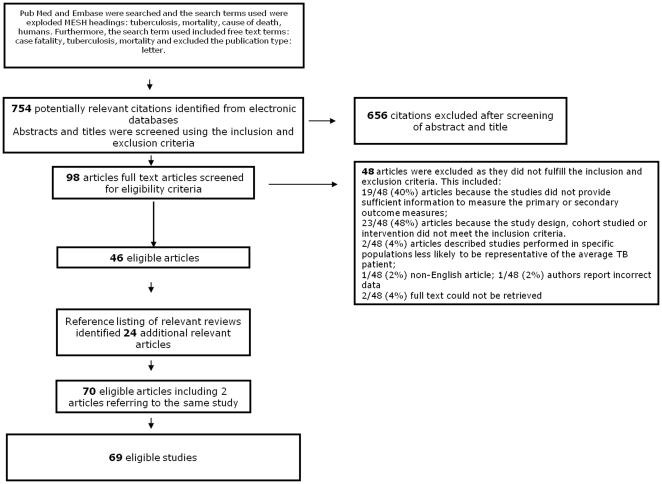
Flow diagram of papers accepted and rejected during selection procedure.

Seven - hundred-fifty-four potentially relevant studies were identified from the PubMed and Embase databases of which 46 were eligible for our review. Reference listing of relevant review articles identified 24 additional articles See figure 1 for the flow diagram for study selection.

### Description of characteristics included studies

The search identified 70 relevant articles of which two articles referred to the same study but were included separately in the analyses as one article provided data on mortality due to TB [25] and the other article provided data on mortality during and after TB treatment [26]. Twenty-eight of the 69 different studies were prospective cohort studies [23], [25]–[52]; 31 retrospective cohort studies [24], [53]–[82], five retrospective record reviews [83]–[87]; four trials [88]–[91] and one case control study [92]. (Table S1)

Almost half of the studies (46%, 32/69) were performed in Africa [27]–[35], [37]–[40], [42], [48]–[50], [52], [58], [61]–[63], [69], [74], [81], [82], [86]–[91] followed by 18 studies in Asia [23], [36], [43]–[47], [60], [64], [65], [70], [71], [73], [75], [76], [78]–[80]; nine studies in America [24]–[26], [54], [57], [72], [77], [83]–[85] and ten European studies [41], [51], [53], [55], [59], [66]–[68], [73], [92] and one study from Australia [56]. These numbers include the study results in six different countries as reported by Espinal *et al.* [73] and the study of Ciglinecki *et al.* [89] reporting study results of two different African countries.

The geographical coverage of the study population was in 26 studies one city including its surrounding area's [28]–[32], [35], [37]–[39], [45], [47], [48], [50], [54], [55], [57], [65], [67], [73], [76], [85]–[89], [91] in 25 studies one specific district or region in the country [25]–[27], [33], [34], [42], [43], [46], [49], [53], [58]–[63], [69]–[74], [80], [82], [90], [92], 10 national studies [36], [41], [51], [64], [66], [68], [77], [79], [81], [84] although one [51] reported that national coverage was not 100%; 8 were done in states or provinces [23], [24], [40], [52], [56], [75], [78], [83] and one study was performed in hospitals covering 80% of the population [44].

Eighteen studies did not report on age of the TB patients. In the majority of the included studies that reported on age of the TB patients initiating TB treatment TB patients were between 20 and 40 years old. Hussey *et al.* [86] included children (median age 10.5 months) with childhood miliary tuberculosis while the studies of Fielder *et al.* [57]; Shen *et al.* [76] and Sakurai *et al.* [47] included a relative large proportion of individuals aged older than 50 years. (Table S1)

Only 5 of the 32 studies reporting on mortality in TB patients with HIV specifically reported the use of prophylaxis in HIV infected TB patients [23], [54], [82], [90], [91]. while one study reported that no prophylaxis was used [89]. Use of antiretrovirals in HIV infected TB patients was reported in 5 studies (16%) [23], [53]–[55], [91] of which 3 were conducted after 1996, when highly active antiretroviral therapy (HAART) [23], [55], [91] became available. Only the study of Abdool-Karim *et al.* [91] reported that 100% of the HIV infected TB patients received antiretroviral treatment; while in the study of Cain *et al.* [23] and Dean *et al.* [55] respectively 44% and 46% of HIV infected TB patients received ARTs.

Only a few studies included detailed information on the severity of HIV disease by for example providing information on CD4 cell count [30], [32], [53], [54], [90], [91] or HIV stages [32], [38], [50], [54], [89], [91]. In the majority (83%) of the studies reporting on CD4 cell count either the median CD4 cell count was <200 cells/mm^3^ [91], [93] or at least 50% of the cohort had CD4 cell counts less than <200 cells/mm^3^ [32], [53], [90]. In the three studies not reporting CD4 cell count but only reporting on HIV stage according to WHOs clinical AIDS definition [38], [50], [89] the percentage of HIV positive TB patients defined as having AIDS varied from 61.8% [38] to 76% [50]. Comorbidities were reported in less than one third (27%) of the studies reporting on mortality in HIV positive TB patients [23], [28], [29], [37]–[39], [50], [55], [90]. Due to lack of detailed information regarding use of antiretrovirals, severity of HIV disease and comorbidities we did not further include these parameters in any subgroup analyses.

### Primary outcome measure: Mortality due to TB

Cause of death was reported in 22 studies [23]–[25], [30], [44], [47], [50], [53], [54], [56], [57], [59], [66]–[68], [75], [76], [79], [83]–[85], [90]. Use of verbal autopsy data additional to clinical information was reported by two studies [23], [90]. Twenty studies established cause of death using clinical records, review of files and/or death certificates. In these studies, TB was either reported as ‘primary cause of death’ [25], [54], [56], [57], [84] or as ‘cause of death’ [44], [47], [66]–[68], [75], [76]; while studies also reported TB as ‘contributory cause of death’ [30], [56], [57], [76], [84], [85], attributed to TB [79], ‘immediate cause of death’ [50] or ‘related to TB’ [24], [53], [59], [83]. Data of the primary and contributory cause of death were aggregated per study in three studies distinguishing between TB as primary and contributory cause of death [56], [57], [84]. Three studies did not report on which was the primary cause of death when TB was recorded as contributory cause of death. Three studies reported on mortality in new TB patients [30], [44], [57]; while only one study [59] provided mortality data in retreated TB patients and the majority of studies did not reported separately for new and retreated TB patients.

**Figure 2:**
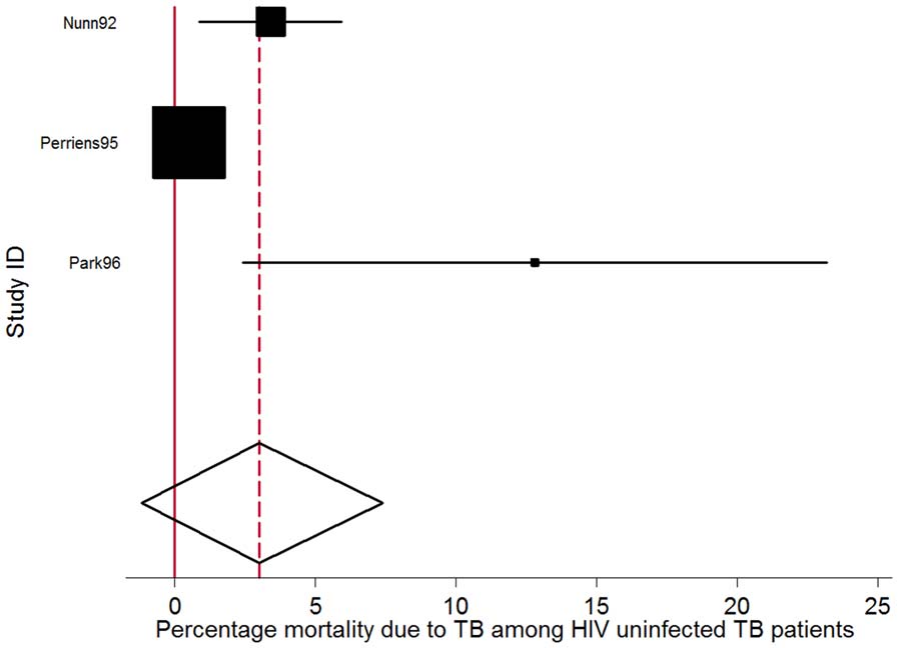
Forest plot of 3 studies reporting mortality *due* to TB as percentage of total number of HIV uninfected persons receiving TB treatment. (I-squared = 78.6%, p = 0.009). The Study ID on the Y-axis includes the name of the first author and publication year; for each study the central square indicates the mortality percentage and the horizontal line denotes the 95% confidence interval (CI) around the mortality percentage. The size of the square indicates the impact the specific study has on the point estimate of the pooled estimate. The vertical dashed line indicates the pooled mortality percentage and the outer edges of the diamond represent the 95% confidence interval (CI) around the pooled estimate; the X-axis indicates the scale of mortality percentage.

**Figure 3:**
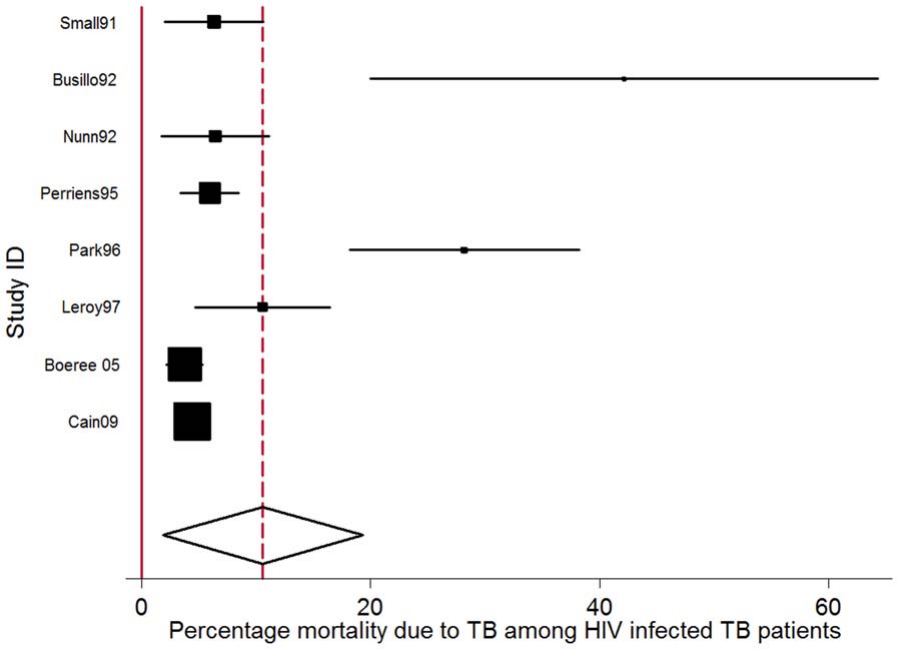
Forest plot of 8 studies reporting mortality *due* to TB as percentage of total number of HIV infected persons receiving TB treatment. (I-squared = 82.1%, p = 0.000). The Study ID on the Y-axis includes the name of the first author and publication year; for each study the central square indicates the mortality percentage and the horizontal line denotes the 95% confidence interval (CI) around the mortality percentage. The size of the square indicates the impact the specific study has on the point estimate of the pooled estimate. The vertical dashed line indicates the pooled mortality percentage and the outer edges of the diamond represent the 95% confidence interval (CI) around the pooled estimate; the X-axis indicates the scale of mortality percentage.

The pooled TB CFR (percentage death *due to* TB) in HIV-uninfected persons was 3.0% (95% CI: −1.3%–7.4.%) and the pooled TB CFR in HIV infected persons was 9.2% (95% CI: 3.7%–14.7%), based on the results of three and eight studies respectively that reported on cause of mortality in these groups (Table 1, Figure 2&3). The percentage deaths due to TB in smear positives (11.7% 95% CI: 7.4%–15.9%) was higher than in smear negatives (5.7%, 95% CI: 2.6%–11.1%, irrespective of HIV status, based on the results of 3 and 2 studies respectively (Table 1). Only two studies [54], [85] provided information on TB as cause of death for multi drug resistant (MDR) TB patients. The pooled TB CFR in HIV positive MDR TB patients was 30.9% (95%CI: 21.7%–40.1%).

**Table 1:** Average pooled tuberculosis (TB) Case Fatality Ratio (CFR) (expressed as percentage) estimated from studies that have registered TB as cause of death * in treated TB patients (primary endpoint) or studies that have reported mortality during TB treatment ‡ (secondary endpoint).

	Primary endpoint		Secondary endpoint	
	CFR (%) (95% Confidence Interval) §	References	CFR (%) (95% Confidence Interval	References
*Overall*	4.2% (2.3%–6.1%)†	[24], [47], [56], [66]–[68], [75], [76], [79], [84], [85]	5.8% (3.1%–8.4%)	[41], [51], [56], [58], [60], [62], [63], [66], [68]–[70], [72], [73], [75], [76], [78], [80], [92] ≠
*HIV uninfected*	3.0% (0.0%–7.4%)†	[30], [50], [85]	3.5%% (2.0%–4.9%)	[27]–[30], [32], [35], [37]–[40], [48], [49], [51], [69], [74], [77], [78], [87], [89]
*Smear positive*	0.5% (0.0%–2.9%)	[30]	1.6% (0.1%–3.2%)	[29], [30], [32], [33], [38], [40], [89]
*Smear negative*	–	–	7.5% (−0.9%–16.0%)	[33], [40], [74]
*HIV infected*	9.2% (3.7%–14.7%)†	[23], [30], [50], [53], [54], [83], [85], [90]	18.8% (14.8%–22.8%)	[23], [27]–[30], [32], [35], [37]–[40], [48], [49], [51], [55], [63], [69], [74], [77], [78], [82], [83], [87]–[91]
*Smear positive*	4.6% (3.2%–6.1%)	[30], [90]	15.6% (11.1%–20.2%)	[29], [30], [32], [33], [38], [40], [63], [74], [88]–[91]
*Smear negative*	–	–	37.7% (22.7%–52.7%)	[33], [40], [63]
*Smear positive*	11.7% (7.4%–15.9%)	[25], [57], [59]	8.3% (5.9%–10.7%)	[26], [27], [31]–[33], [36], [38], [40], [42], [43], [45], [46], [52], [57]–[59], [61], [63], [64], [71], [74], [76], [81], [89], [91], [92]
*Smear negative*	5.7% (2.6%–11.1%)‡	[44], [59]	15.2% (7.5%–22.8%)	[27], [31], [33], [34], [40], [42], [59], [63], [68], [69], [76], [81], [86] ¶
*Extra Pulmonary*	included in overall smear negative CFR	included in overall smear negative CFR	18.2% (9.6%–26.7%)	[33], [42], [63], [65], [68], [81], [86]
*Multi drug resistant TB*	21.1% (14.6%–27.5%)	[85]	4.7% (0.2%–9.2%)	[60], [73], [76] ‡
*HIV infected*	30.9% (21.7%–40.1%)	[54], [85]		
*HIV uninfected*	12.8% (2.3%–23.3%)	[85]		
*Smear positives*			12.2% (2.2%–22.2%)	[26]

* Studies have registered cause of death based on either verbal autopsy additional to clinical information [23], [90] or vital registration in which clinical records and/or death certificates have been reviewed and TB was either reported to be (primary) cause of death [25], [54], [56], [57], [84] [44], [47], [66]–[68], [75], [76]; contributory cause of death [30], [56], [57], [76], [84], [85]; attributed to TB [79], TB as ‘immediate cause of death’ [50] or death was described as ‘related to TB’. [24], [53], [59], [83];

‡ Including one study {326/id} reporting treatment outcome data from 6 individual studies in 6 different countries and one study reporting on two separate cohorts in Zambia and Malawi [89] The individual estimates in these two studies have contributed to the pooled estimates as reported in this table;

§ Results of individual studies which have published TB mortality data with different certainties of TB as cause of death [56], [57], [84] have only been included once in the overall analyses by calculating the cumulative mortality of both categories of TB as cause of death;

† Including two studies in only MDR TB patients [54], [85];

‡ Including one study in only extra–pulmonary TB cases [44];

≠ Including one study in only MDR TB patients [60];

¶ Including seven studies reporting mortality during TB treatment of smear negative pulmonary TB patients [31], [34], [40], [42], [63], [76], [81]; three studies reporting mortality during TB treatment in both smear negative pulmonary TB and extrapulmonary TB [27], [33], [59]and three studies reporting mortality during treatment of extra pulmonary TB patients. [68], [69], [86].

Excluding the two studies in HIV infected TB patients in which treatment was either classified as non-standardized [85] or in which some patients received non standardized and some standardized TB treatment [23] only slightly decreased the pooled CFR from 9.2% to 8.4% (95% 3.0–13.7%). Including only studies conducted in non-MDR TB patients receiving standardized TB treatment [30], [50], [53], [83], [90] further decreased the CFR to 5.8% (95% CI: 3.9%–7.8%).

All three studies in HIV uninfected TB patients received standardized TB treatment, but excluding the study done in HIV uninfected MDR TB patients [85] resulted in a CFR of 1.6% (95% CI −0.4%–3.7%). The smear positive TB patients [25], [57], [59] all received standardized treatment and did not specifically report that MDR TB patients were included. In the sub group analyses of smear negative TB, excluding the study of Lau *et al.* [44] in which extra pulmonary TB patients received non standardized treatment, the CFR based on only 1 study [59] was 2.9% (95% CI: 1.9%–4.0%).

### Secondary outcome measure: Mortality during TB treatment

Mortality *during* TB treatment was reported in 59 studies [23], [26]–[43], [45], [46], [48], [49], [51], [52], [55]–[66], [68]–[78], [80]–[83], [86]–[92]. Eight studies provided mortality data in either retreated [26], [33], [51], [60], [76], [91] or relapsed TB patients [27], [59] while the majority of the studies reported mortality data on either only new TB patients or new and retreated TB patients. In 6 studies, the treatment strategy was categorized as non-standardized treatment [41], [60], [65], [75], [77], [78] including 4 studies in which the treatment strategy was not specified [41], [75], [77], [78]. The study of Cain *et al.* [23] described that the cohort included both persons receiving standardized and non standardized treatment. These six studies included either only HIV infected - and/or HIV uninfected TB patients [23], [77], [78], MDR TB patients [60], extrapulmonary TB patients [65] or did not report stratified results [41].

**Figure 4:**
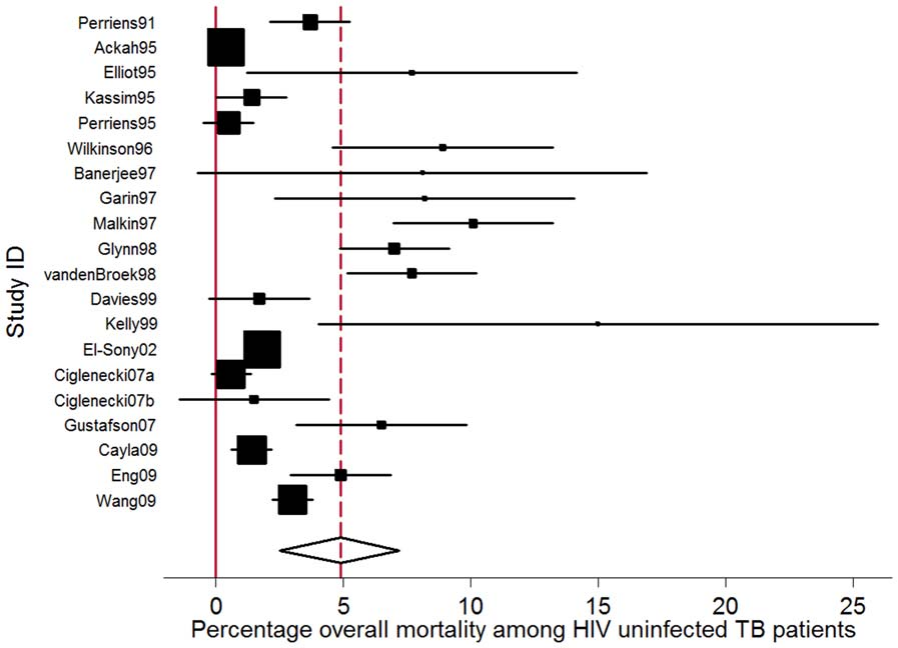
Forest plot of 19 individual studies reporting mortality *during* TB treatment as percentage of total number of HIV uninfected persons receiving TB treatment. (I-squared = 88.6%, p = 0.000). The Study ID on the Y-axis includes the name of the first author and publication year; for each study the central square indicates the mortality percentage and the horizontal line denotes the 95% confidence interval (CI) around the mortality percentage. The size of the square indicates the impact the specific study has on the point estimate of the pooled estimate. The vertical dashed line indicates the pooled mortality percentage and the outer edges of the diamond represent the 95% confidence interval (CI) around the pooled estimate; the X-axis indicates the scale of mortality percentage.

**Figure 5:**
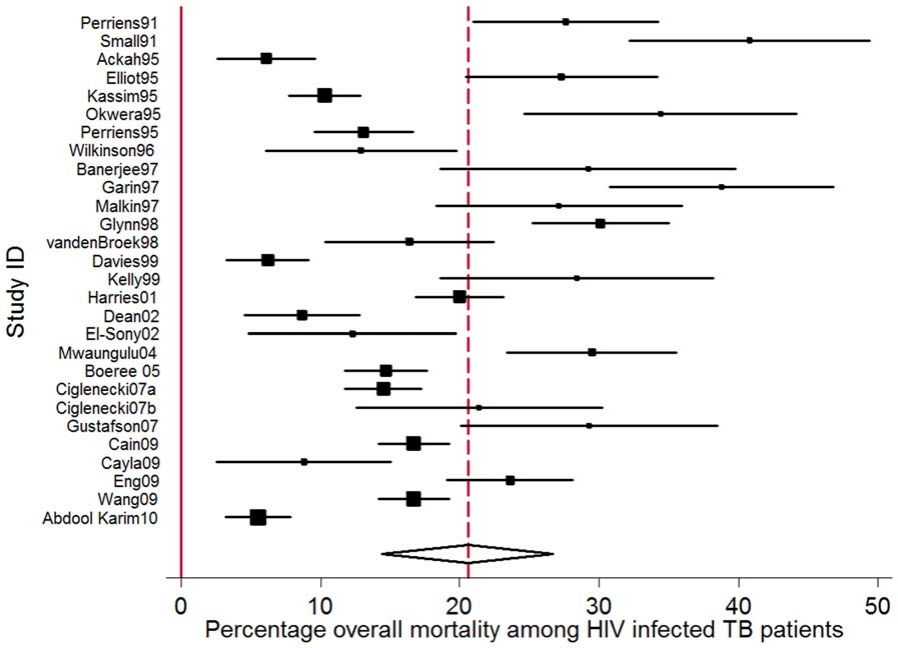
Forest plot of 27 individual studies reporting mortality *during* TB treatment as percentage of total number of HIV infected persons receiving TB treatment. (I-squared = 92.8%, p = 0.000). The Study ID on the Y-axis includes the name of the first author and publication year; for each study the central square indicates the mortality percentage and the horizontal line denotes the 95% confidence interval (CI) around the mortality percentage. The size of the square indicates the impact the specific study has on the point estimate of the pooled estimate. The vertical dashed line indicates the pooled mortality percentage and the outer edges of the diamond represent the 95% confidence interval (CI) around the pooled estimate; the X-axis indicates the scale of mortality percentage.

Stratified by HIV status, the pooled percentage of deaths *during* TB treatment was 3.5% (95% CI: 2.5%–7.2%) in HIV uninfected persons and 18.8% (95% CI: 14.8%–22.8%) in HIV infected persons, based on the results of 19 and 27 individual studies respectively (Table 1, Figure 4&5). The percentage of deaths in smear positives was 8.3% (95% CI: 5.9%–10.7%) and 15.2% (95% CI: 7.5%–22.7%) in smear negatives based on the results of 27 and 13 studies respectively. (Table 1) Excluding the studies with non standardized TB treatment [23], [77], [78] resulted in similar CFRs for HIV uninfected persons (3.3%; 95% CI: 1.7–5.0) as well as for HIV infected persons (18.8%, 95% CI: 14.0%–22.6%). Excluding the three studies conducted in HIV infected TB patients after 1996 and reporting that at least part of the cohort received ARVs resulted in a slightly higher CFR (20.4%; 95% CI: 16.2%–24.6%) but the 95% CIs were overlapping.

#### Proportion of deaths due to tuberculosis among treated tuberculosis patients that died during tuberculosis treatment

Not all deaths occurring *during* TB treatment have TB disease as their cause. In our systematic review, 13 studies provided information on the total number of TB patients dying *during* TB treatment as well as the total number of TB patients dying *during* TB treatment *due to* TB (Table S2) [23], [30], [50], [56], [57], [59], [66], [68], [75], [76], [83], [85], [90]. Using these studies we assessed the proportion of deaths *during* TB treatment that were *due* to TB.

Excluding the study with HIV infected MDR TB patients [85], the average proportion of HIV infected persons dying *during* TB treatment was 18.9% (95% CI: 11.6%–26.2%). In these studies among HIV infected persons dying during TB treatment only 4.6% (95% CI 3.2%–6.0%) of the deaths were reported to be *due to* TB [23], [30], [50], [83], [90]. Consequently, based on these results we estimate that of all HIV infected TB patients dying during TB treatment around 24% die due to TB. Cause of death in HIV infected TB patients was assessed by different methods or a combination of methods, review of clinical data, autopsy results, death certificates, medical records and interviews with relatives (Table S2). Other main causes of death as reported in these studies included *Pneumocystis carinii* Pneumoniae [83], pneumonia [50], [83] gastro intestinal disease [50], [90] wasting syndrome, Kaposi's sarcoma [83], [90], meningitis [90], central nervous system syndrome not caused by infections, (other) opportunistic infections [83], [90], toxic epidermal necrolysis [50] and miscellaneous causes [83] or unknown cause [50], [83], [90].

Excluding the study with HIV uninfected MDR TB patients [85], the average proportion of HIV uninfected TB patients [30], [50] and TB patients in countries of which we assume have a low HIV prevalence [56], [57], [59], [66], [68], [75], [76], [94], dying *during* TB treatment was 6.5% (95% CI: 3.0% to 9.9%, while among these studies 3.4% (95% CI: 1.1% to 5.7%) died *during* TB treatment *due* to TB. Consequently, it seems that of all HIV uninfected persons dying during TB treatment, about one half die *due* to TB.

Information on timing of mortality due to TB was only reported in six studies [23], [50], [56], [59], [83], [84] The majority of deaths during TB treatment that were due to TB appeared to occur in the first months after TB diagnosis or start of treatment. Small *et al.* [83] reported that 75% (6 of 8) of TB patients dying due to TB died within the first month of TB treatment. Mathew *et al.* [59] reported that at least 65% of the persons who died due to TB died within the first 3 months after initiating TB treatment. Walpola *et al.* [56] reported that of the 54 TB related deaths that started TB treatment 43% died within one month and 74% within 3 months after commencing TB treatment, similar to the study of Xie *et al.* [84] where 67% died within 3 months after initiating TB treatment. In the study of Cain *et al.* [23] also 65% of the deaths of which probability of TB cause of death was probable or high died within 2 months after initiating TB treatment. Nunn *et al.* [50] looked at TB as cause of death within 6 months after initiating 12 month duration treatment. Among HIV infected- and HIV uninfected persons 57% (4 of 7) and 83% (5 of 6) of all TB deaths within six months of follow up after TB treatment was initiated, occurred in the first month.

#### Overall mortality within four years after initiating TB treatment

Deaths occurring after completion of TB treatment may also be attributed to TB and therefore be included in the TB CFR. Fourteen studies reported follow–up data on overall mortality after the duration of the TB treatment or referred to other publications of the same study that described this follow up data [26]–[29], [33], [38], [43], [45], [46], [48], [70], [83], [87], [89] (Table S3). The average percentage of HIV uninfected persons dying within a maximum follow up period of four years after initiating TB treatment was 9.0% (95% CI: range 4.5% to 13.5%) and 41.7% (95% CI: 30.8% to 52.5%) among HIV infected TB patients. For this analysis we excluded the study of which follow up period was unknown [26]. Among HIV uninfected persons an average 46% (95% CI: 30.7% to 62.2%) of total deaths within maximum four years of follow up died *after* TB treatment completion [27]–[29], [33], [38], [48], [83], [87], [89]. This indicates that around 54% of all deaths in HIV uninfected TB patients died during TB treatment. Among HIV infected persons in the same studies this percentage was 41.3% (95% CI: 31.2%–51.3%).

It should be noted that of the 14 studies containing data on mortality during TB treatment and mortality during the follow up period, only three studies reported mortality data due to TB [27], [45], [83]. The percentage of deaths occurring after completion of TB treatment that could be attributed to TB varied largely from 10% [83] to 80% [45].

## Discussion

We have conducted this systematic literature review to estimate the TB case fatality ratio in TB patients starting TB treatment and consequently contribute to a revision of the CFR for TB patients currently used by WHO for estimating TB mortality [3], [6], [11]. We found that among HIV infected persons initiating TB treatment 9% (95% CI: 4% to 15%) and among HIV uninfected persons 3% (95% CI: −1.2% to 7.4%) died *due to* TB during the follow up period of the study. Because of different follow up times of the studies included we were unable to establish the time period to these percentages. The number of studies specifically reporting on TB as cause of death was limited therefore we also looked at studies reporting on *overall* mortality during TB treatment. We found that among HIV infected persons starting TB treatment 19% (95% CI: 15%–23%) died *during* TB treatment and among HIV uninfected persons this percentage was 4% (95% CI: 2%–5%).

Previously, WHO has published CFRs which were partly based on estimations of the percentage of TB patients dying *from* TB during TB treatment. The results of our systematic review add data for the underlying assumptions. We found that of the total number of deaths during TB treatment, TB had been considered the cause of, or contributed to, death in 24% of the deaths in HIV infected persons and in about half of deaths in HIV uninfected TB persons. During routine TB treatment outcome monitoring, reported deaths during TB treatment usually do not discriminate between deaths due to TB and deaths due to other causes. Therefore, our information may be useful for linking with routine reporting of deaths during TB treatment and estimate the percentage of deaths due to TB when overall mortality during TB treatment is reported.

A few limitations need to be considered. First, the average proportion of deaths due to TB among all deaths during TB treatment are based on only five studies in HIV infected - and ten in HIV uninfected TB patients. In the individual studies this proportion varied from 16% in the USA in which 41% of the HIV infected population starting TB treatment died during TB treatment [83], to 46% in the study of Perriens *et al.*. [30] in which 13% died during TB treatment. This wide inter study variation may be due to variations between settings e.g. as a consequence of differences in age of the TB population in specific countries and potential bias and confounding factors. The percentages are also sensitive to miscoding causes of death. According to the International Classification of Diseases, 10^th^ revision (ICD–10) deaths from TB in individuals infected with HIV should be classified as HIV deaths [6]. In some countries TB might be coded as contributory cause of death [2]. Several definitions were used in the studies. Leroy *et al.* [53] classified deaths in people living with HIV and TB as definite TB death in patients without a history of other AIDS–related disease and without any other known cause of death. Walpola *et al.* [56] reported TB as primary and contributory cause of death but did not differentiate between HIV infected and HIV uninfected TB patients. Diagnosing TB as cause of death is known to be problematic in several settings. Moorman *et al.* [95] conducted a study to assess the quality of data of reported deaths from TB in a rural South African district hospital, and determined the actual cause of death of patients who died whilst on TB treatment. They found several reasons for miscoding of TB deaths; e.g. TB was assumed to be the cause of death if the patient died on the TB ward. Furthermore, death certificates were inaccurate and often two illnesses were recorded as the main cause of death. Older patients over the age of 65 were more likely to have a number of concomitant diseases that may cause death, and particular care should be taken in this age group in recording a death as due to TB. Despite uncertainties in measuring TB mortality, causes of death as coded in the death certificates are still considered as the gold standard. By means of adequate training of medical doctors filling and signing the death certificates and the use of standardized coding practices, these uncertainties can be minimized. To obtain a more accurate estimate of TB CFR in England and Wales, Crofts *et al.* [96] compared mortality ascertained by the national TB surveillance with record linkage to mortality information from the National Health Service central register and death registration from the Office for National Statistics. A capture recapture methodology was used to estimate unascertained deaths for final calculation of CFR. They not only quantified the number of deaths missed by the national TB surveillance, but their findings also showed consistently that half of deaths among TB patients are due to TB.

The CFRs estimates as published by WHO previously were based on assumptions regarding the percentage of late deaths from relapse or post–tuberculous complications and deaths of untreated TB patients [6], [11]. The study of Vijay *et al.* showed that 66% of the deaths occurring within 2.5 years after initiating TB treatment occurred among the defaulters. Of those deaths, 87% were considered attributable to TB [45]. None of the included studies showed data on TB mortality in patients who defaulted, transferred out or were lost to follow up. We only found three studies [27], [45], [83] reporting on a wide variation of mortality *due* to TB after treatment completion. The limited data on mortality after TB treatment completion and/or among defaulters and lost to follow up, prevented us from making firm conclusions about the percentage of deaths after completion of TB treatment. Previously, Korenromp *et al.* [11] showed that across six studies tracing defaulters and transfers out over a median of one year after start of treatment, a median of 21% (range 8% to 33%) of the defaulters and transfers out had died increasing the total percentage of overall deaths by 3.5%. The results of these six studies may imply that an additional 3.5% should be used to account for the proportion of overall deaths among TB patients initiating treatment who defaulted or transferred out. However, the percentage of defaulters and patients with undocumented outcomes varies widely between settings [10], [97]. An additional factor may be added for mortality among those people lost to follow up and might have died because of TB.

The true TB CFR includes the assessment of mortality in not only diagnosed TB patients undergoing TB treatment, but also in undiagnosed and consequently untreated TB patients. Our systematic review did not assess the percentage of deaths among undiagnosed TB patients, which may constitute a major contribution to global TB burden.

### Generalisability

In our review, we have included both observational and experimental studies. Differently from the usual high-quality found in randomised trials, findings of observational studies are often distorted by confounding and selection bias. Our primary outcome of interest was mortality *due* to TB and our search strategy was specifically set up to answer that question. Consequently, we were not specifically interested in TB treatment trials, although our search resulted in four TB treatment trials [88]–[91] of which 1 trial reported on mortality among HIV infected *due* to TB [90].; two trials on mortality among HIV infected during TB treatment [88], [91] and one trial included HIV uninfected TB patients [89]. This small number prevented us from conducting separate analyses for observational and experimental studies. Studies are often conducted in populations in which the researchers have a special interest. For example studies on the cause of death may be conducted in populations with a high death rate. We have excluded studies among populations that cannot be considered representative for the general population. If we would have included studies conducted in populations that are ‘special’, then the results of our meta-analysis would have been less generalisable.

The majority of the studies were performed in one district, city, surrounding or province which may have reduced the representativeness of the results to the nation. The variation in TB CFR between studies may have been influenced by unmeasured factors or may partly be explained by the differences in the data analyses performed in these studies. The unmeasured factors include, for example, the quality of the TB program, changes in national DOTS coverage, treatment regimen, duration of HIV epidemic in the area, completeness of information on smear and HIV status and severity of active TB lesions at study entry [47]. In some prospective studies the data were analyzed as cross–sectional studies by not taking into consideration the loss to follow up as they only limited the analyses to the group of people with known data, in contrast to other studies that either reported that loss to follow up was either excluded or included in the denominator. These factors may have influenced the CFR in both directions. Despite the observed heterogeneity we have chosen to pool the results of the individual studies. For all analyses we used a random effect model allowing studies to come from different source populations with variation in CFR [98]. The overall subgroup analyses based on HIV and smear status included all more than 10 studies. Excluding the studies with non standardized TB treatment did not result in different CFR, indicating that the overall results were robust. To reduce heterogeneity we also presented the results excluding the studies that only included MDR TB patients as this largely contributed to heterogeneity. To assess whether the results in HIV infected persons can be generalized to populations of varying degrees of HIV-related immunosuppression we were limited by the fact that the majority of the studies focused on TB cohorts without providing detailed information on severity of HIV disease. Those studies reporting on HIV status showed that the majority of HIV infected TB patients had a similar degree of immunosuppression as indicated by a CD4 cell count <200 cells/mm^3^. However from the majority of studies this information is lacking. To make a strong statement on generalizing our results to HIV infected populations receiving highly active antiretroviral therapy (HAART) we again faced the limitation of lack of detailed information. Use of antiretrovirals was only reported in 16% of the studies conducted in HIV infected TB cohorts of which only 3 studies where conducted after 1996, the time period when HAART became available [55], [91], [99]. Excluding these studies from the overall analyses in HIV infected persons did not had a significant effect on the CFR. This may be due to the fact that the other studies may also have included HIV infected persons receiving HAART but failed to report this and masking a difference. Therefore we conclude that the results of this meta-analysis cannot be applied to populations receiving ART. To date, WHO has not separated ART cohorts from non ART cohorts, mostly because the vast majority of TB patients infected with HIV were not on ART as of 2009 (personal communication). Previously, we found that in a cohort of people living with HIV exposed to HAART the presence of TB did not result in an increased risk of dying compared to those HIV infected persons not having TB [100]. In this study we were able to pool multivariate hazard ratio's of the individual studies, thereby reducing potential confounding. However in the current meta-analysis heterogeneity between the studies and the lack of information on potential confounding factors may have biased the results. We could not stratify for different age categories but the majority of cohorts in our meta-analysis included cohorts aged between 20–40 years which is also the most frequent reported age group of most reported cases worldwide [1], [7]. Therefore we feel our results are fairly representative regarding age of the most TB patients.

For several other subgroups we identified only a small number of studies. It is difficult to draw conclusions among such a limited number. The pooled results of the subgroup analyses presented in Table 1 that only include a small number of studies should be cautiously interpreted until the results of more studies become available. For example: we found that smear negative TB patients infected with HIV seemed to have a higher CFR (38%; 95% CI: 23%–53%) than smear positive TB patients infected with HIV (19%; 95% CI: 15%–23%). Only three studies provided mortality data in the subgroup of HIV infected smear negative TB patients, consequently we think it is too early to make a strong statement that this difference is indeed true.

### Assessment of publication bias

We aimed to reduce publication bias by not only identifying studies in Pubmed and Embase databases but also conducting reference listing of relevant reviews either found in the search strategy or known by experts. Limitations of our search strategy however, included the exclusion of non-English publications and we did not conduct hand searches of journals as there were not available to us. As we included observational studies without for example assessing an effect of an intervention or success of a diagnostic tool by comparing exposed and non-exposed cohorts, we cannot state with certainty which direction this would have influenced our results. In studies assessing an effect, the studies showing a negative effect may not have been published [101] while it has also been shown previously that authors were more likely to publish statistically significant results in English literature compared to their national language [102]. It may be possible that studies including a large number of deaths and other negative outcomes have not been published resulting in a possible underestimation of TB mortality. Consequently, publication bias may have influenced the results of our study but this may be limited especially because our main outcome of interest was mortality within a TB cohort, which did not include a comparison with a measure of effect.

### Conclusions

In conclusion, despite the limitations as described earlier the results of this systematic literature review allowed us to quantify TB CFR in the main overall subgroups defined on HIV status and smear status. Mortality percentages in HIV infected and HIV uninfected due to TB and mortality percentages during TB treatment assessed from this literature review, were used by WHO to define prior distributions of CFR in countries with vital registration systems in a Bayesian model that generated posterior distributions of CFR. The posterior distributions were then used to predict mortality in countries with no functional vital registration system. Separate models were used in the group of high–income countries and in the group of Eastern European countries (where CFR are higher, partly due to the high burden of MDR–TB) [103].

Uncertainty still remains significant because we could not retrieve sufficient data on the following parameters: 1) the percentage of deaths from TB among TB patients dying during TB treatment; 2) the percentage of deaths from TB in TB patients lost to follow up, relapse cases and defaulters; 3) the percentage of deaths from TB after completion of initial treatment. To obtain this data, in depth analyses of National Tuberculosis Program data are needed. Preferably data analyses of those programs where HIV testing takes place and where adequate follow up and/or possibility to ascertain data on TB deaths and number of deaths in defaulters is in place. Furthermore, the use of anti retroviral therapy should be documented to quantify the CFR in HIV infected TB patients and the role of ART. For estimation purposes CFRs need to be adjusted for ART, but this meta-analysis does not provide data to estimate the adjustment factor. It is important to improve and validated the measurement of TB mortality.

Operational studies should ensure the quality of data collection, including collection of information about cause of death and variables related to this, can often be done more rigidly in a clinical trial design compared to other study designs. So using the data of a clinical trial to better evaluate the cause of death in TB patients can provide good information. Strengthening of TB surveillance system could include the possibility to discriminate between deaths due to TB and deaths with TB and follow up of TB patients even after treatment completion or default. This can only be established if patients reporting for retreatment are clearly classified and the new record of the retreatment episode is linked to previous treatment records. Following these recommendations the gap in knowledge can be addressed and improving our understanding of the magnitude and time trends in the burden of TB disease.

## Supporting Information

Table S1 * Unless specified otherwise the mean and range are presented; § Clarification for abbreviations and symbols used table: TB = tuberculosis, PTB = pulmonary TB; ETB = extra pulmonary TB; Sm = smear; + = positive; − = negative; +/− = data stratified for positive and negative; all = no stratification; n/a: not applicable because smear diagnostic did not take place; unk = unknown; HIV = Human Immunodeficiency Virus; ± Total number of deaths *during* TB treatment unless specified otherwise, data in subgroups (e.g. HIV status or smear status) are used in the specific subgroup analyses; † deaths *due* to TB; ≠loss to follow up includes not only persons ‘lost to follow up’ but also ‘transfer outs’ and ‘default’ if these numbers are presented because mortality in these persons is unknown; n.r.: not reported, because only reporting about people with known outcome, missing data have already been excluded from the analyses; – Loss to follow up is unknown and unknown if denominator in–or excludes ltfu;** Numbers are not excluded from denominator; Follow up period is either during TB treatment (tbx); expressed in person years (py); or unknown (unk). 1: HIV testing was accepted by 110 (54%) of 205 patients; 2: These 2 articles refer to the same study but one articles contributes to information for the primary outcome measure [25] and the other for the secondary outcome measure [26] 3 Included the countries Republic of Korea, Peru, China, Russia, Dominican Republic and Italy; 4 Deaths among people who survived first two months after initiating TB treatment; 5: We only included the group of patients with treatment initiated after the war.
(DOC)

Table S2 * MDR = multidrug resistant TB; PTB = pulmonary TB; **^†^** TB at least a major contributor to death in HIV– positive individuals; ‡ Of the 42 patients who died, tuberculosis was listed on the death certificate as the primary cause of death in 16 (38%), as a contributing cause of death in 15 (36%), TB was considered a major contributing cause of death for 4 deaths during the 6–month treatment period, 16 deaths were considered to be related to TB because they occurred before the response to anti TB therapy could be assessed; ** mortality during first 6 months of 12 months treatment duration; **^††^** TB was recorded as immediate cause of death if death occurred within one month after initiating TB treatment and there was no other recorded causes. In patients with multiple cause of death the immediate cause was determined by review of the clinical data and the establishment of consensus. **^‡‡^** Autopsy results were available from 55 of 63 deaths. For 5 of 63 TB deaths HIV status was undocumented while 58 deaths were HIV-. These 63 TB deaths include 45 smear+ TB deaths.
(DOC)

Table S3 * Categorization subgroups: Human Immunodeficiency Virus positive (HIV pos.) or negative (HIV neg.); Smear positive (Sm pos.) or negative (Sm neg.); **†** Unless specified otherwise follow up time was calculated from the initiation of TB treatment; **§** Unless specified otherwise, mortality is assessed as percentage of original TB cohort initiating TB treatment without adjustment for percentage of patients lost to follow up; ^1a^ 999,7 person years (p.yrs) follow up time for HIV pos. and 560 p.yrs follow up time for HIV neg., ^1b^ at 12 months 11.7% loss to follow up (ltfu); ^2a^ 113,4 p.yrs follow up time for HIV pos. and 123,1 p.yrs follow up time for HIV neg., ^2b^ at 12 months 6% ltfu; ^3a^ 19% (33/174) of HIV pos and 17% (11/65) of HIV neg. was ltfu at end of treatment, ^3b^ 25% (44/174) of HIV pos. and 26% (17/65) of HIV neg. was lftu at the end of follow up period; ^4^ HIV status was available for 96% (793/827) registered TB patients, 11.9% (99/827) registered TB patients was ltfu on 32 months and 18% (152/827) registered TB patients was ltfu at 7 years; ^5a^ at end of 6 months treatment regimen 6.1% (22/359) defaulted, was ltfu or transferred out, and 12% (42/359) was ltfu at end of 2 years follow up, ^5b^ at end of 12 months treatment regimen 21% (63/304) defaulted, was ltfu or transferred out, and 20% (61/304) was ltfu at end of 2 years follow up; ^6^ at end of treatment 5% (195 of 3,600 patients starting TB treatment) could not be followed up because they migrated, transferred out or could not be traced; these are excluded from the denominator in all analyses; ^7^ at the end of treatment 13% (68/512) was loss to follow up and these were excluded from the denominator in all analyses; ^8a^ at end of treatment records were unavailable for 12% (20/170) HIV pos. and 16% (96/597) HIV neg., ^8b^ 39% (67/170) of initial cohort was followed up in HIV pos. and 43% (258/597) in HIV neg.; ^9^ at end of treatment 2% (25/1,171) was ltfu, 46 could not be traced during follow up consequently a total of 6% (71/1,171) was ltfu at end of the study; ^10^ ltfu excluded from the denominator in the treatment outcome analyses; ^11^ at end of treatment 12% (70/561) was ltfu; ^12a^ ltfu excluded from the denominator in the treatment outcome analyses, ^12b^ follow up was only possible for 60% (162/271) of initial cohort.
(DOC)
