# Supplementary material for: Assessing Tuberculosis Case Fatality Ratio: A Meta-Analysis
Source: PLoS One. 2011 Jun 27;6(6):e20755. doi: 10.1371/journal.pone.0020755 (PMC3124477; doi:10.1371/journal.pone.0020755)
Supplement: Table S1 — * Unless specified otherwise the mean and range are presented; § Clarification for abbreviations and symbols used table: TB = tuberculosis, PTB = pulmonary TB; ETB = extra pulmonary TB; Sm = smear; + = positive; − = negative; +/− = data stratified for positive and negative; all = no stratification; n/a: not applicable because smear diagnostic did not take place; unk = unknown; HIV = Human Immunodeficiency Virus; ± Total number of deaths during TB treatment unless specified otherwise, data in subgroups (e.g. HIV status or smear status) are used in the specific subgroup analyses; † deaths due to TB; ≠loss to follow up includes not only persons ‘lost to follow up’ but also ‘transfer outs’ and ‘default’ if these numbers are presented because mortality in these persons is unknown; n.r.: not reported, because only reporting about people with known outcome, missing data have already been excluded from the analyses; – Loss to follow up is unknown and unknown if denominator in–or excludes ltfu;** Numbers are not excluded from denominator; Follow up period is either during TB treatment (tbx); expressed in person years (py); or unknown (unk). 1: HIV testing was accepted by 110 (54%) of 205 patients; 2: These 2 articles refer to the same study but one articles contributes to information for the primary outcome measure [25] and the other for the secondary outcome measure [26] 3 Included the countries Republic of Korea, Peru, China, Russia, Dominican Republic and Italy; 4 Deaths among people who survived first two months after initiating TB treatment; 5: We only included the group of patients with treatment initiated after the war. (DOC) [file pone.0020755.s001.doc]

**Table S1** Characteristics of included studies reporting on mortality in treated tuberculosis patients.

| **Author Year (Reference)** | **Location** | **Design** | **Coverage** | **Study Period** | **Study Size (N)** | **Age (mean, range) ^*^** | **TB type §** | **Sm §** | **HIV §** | **Deaths (n) ±** | **Loss to follow up≠** | **Follow up period** |
| --- | --- | --- | --- | --- | --- | --- | --- | --- | --- | --- | --- | --- |
| **Abdool Karim 2010 [91]** | South Africa | trial | city | 2005-2008 | 343 | 34.4 (19-72) | PTB | + | + | 19 (5.5%) | 32** | tbx |
| **Acka 1995 [32]** | Ivory Coast | prospective cohort | city | 1992–1993 | 460 | 33 (median) | PTB | + | +/- | 12 (2.6%) | 117 ** | tbx |
| **Adatu 2003 [61]** | Uganda | retrospective cohort | district | 1998–1999 | 294 | 30 (median) | PTB | + | unk | 40 (13.6%) | – | tbx |
| **Agutu 1997 [42]** | Somalia | prospective cohort | district | 1994–1995 | 212 | 33% between 25-34 | PTB/ETB | +/- | unk | 15 (7.1%) | 42 ** | tbx |
| **Alavi 2007 [75]** | Iran | retrospective cohort | city | 2002-2006 | 3960 | unk | PTB/EPTB | all | all | 93 (1.0%) † | - | tbx |
| **Banerjee 1997 [74]** | Malawi | retrospective cohort | district | 1995–1996 | 205 | unk | PTB | + | +/- ^1^ | 56 (27.3%) | 15 ** | tbx |
| **Boeree 2005 [90]** | Malawi | trial | district | 1998–2001 | 579 | 32.4 (-) | PTB | + | + | 22 (8.5%) † | 42 ** | tbx |
| **Borgdorff 1998 [66]** | The Netherlands | retrospective cohort | national | 1993–1995 | 4,340 | <25 to > 75 | TB | all | unk | 35 (0.8%) † | 444 ** | tbx |
| **Busillo 1992 [54]** | USA | retrospective cohort | city | 1990–1991 | 19 | 37 (29-61 ) | TB | all | + | 8 (42.1%) † | n.r | unk |
| **Cain 2009 [23]** | Thailand | prospective cohort | provinces | 2005-2007 | 849 | unk | TB | all | + | 38 (4.5%) † | 150** | tbx |
| **Caylà 2009 [51]** | Spain | prospective cohort | national (not 100%) | 2006-2007 | 1490 | > 18 | TB | all | +/- | 27 (1.8%) | 125** | tbx |
| **Ciglinecki 2007 [89]** | Zambia | trial | city | 1996–1999 | 986 | < 35 (50.7%) to > 45 (13.5%) | PTB | + | +/- | 100 (10.1%) | n.r | 598.5 py (tbx) |
|  | Malawi | trial | district | 1996–1999 | 149 | < 35 (35.7%) to > 45 (22.6%) | PTB | + | +/- | 19 (12.8%) | n.r | 91.9 py (tbx) |
| **Conolly 1998 [58]** | South Africa | retrospective cohort | district | 1991–1995 | 2,376 | > 15 | TB/PTB | all/+ | all | 258 (10.9%) | n.r | tbx |
| **Cullinan 1991 [41]** | England, Wales | prospective cohort | national | 1983–1985 | 1,201 | > 15 | PTB | n/a | unk | 137 (11.4%) | 65 ** | tbx |
| **Davies 1999 [49]** | South Africa | prospective cohort | district | 1996–1997 | 416 | HIV+ 34 (s.d.11); HIV- 38 (s.d 15) | PTB | all | +/- | 18 (4.3%) | 6 | tbx |
| **Dean 2002 [55]** | United Kingdom | retrospective cohort | city surroundings & region | 1996–1999 | 183 | 34 (21 – 70) | TB | all | + | 16 (8.7%) | n.r | tbx |
| **DeRiemer 2005 [26]** | Mexico | prospective cohort ^2^ | districts | 1995–1999 | 436 | unk | PTB | + | unk | 22 (5.0%) | - | tbx |
| **Dewan 2004 [92]** | Russia | case control | oblast | 1999–2001 | 1069 | unk | TB | all/+ | all | 63 (5.8%) | – | tbx |
| **Elliot 1995 [29]** | Zambia | prospective cohort | city | 1989–1990 | 239 | unk | PTB | + | +/- | 56 (23.4%) | 44 ** | tbx |
| **El–Sony 2002 [40]** | Sudan | prospective cohort | states | 1998–2000 | 1,797 | HIV-  median 28 (15-49) median HIV+(15-48) | TB/PTB/ETB | all/+/- | all/+/- | 40 (2.2%) | 336 ** | tbx |
| **Eng 2009 [78]** | Cambodia | retrospective cohort | province | 2004-2005 | 1202 | - (2 – 87) | TB | all | all/+/- | 135 (11%) † | 0 | tbx |
| **Espinal 2000 [73]** | Multiple | retrospective cohort **^3^** | national, city, oblast | 1994–1996 | Variable | unk | TB | all | all | variable | variable |  |
| **Fielder 2002 [57]** | USA | retrospective cohort | city | 1993–1998 | 174 | < 49 (51%)  >= 49 (49%) | PTB | +/- | all | 31 (24%) † | 1 | tbx |
| **Garcia–Garcia 2002 [25]** | Mexico | prospective cohort ^2^ | district | 1995–1996 | 443 | median 42 (range 12-97) | PTB | + | unk | 34 (7.8%)† | 0 | median 839 days |
| **Garin 1997 [48]** | Central African Republic | prospective cohort | city | 1993–1994 | 224 | unk | TB | all | +/- | 61 (27.2%) | 0 | tbx |
| **Glynn 1998 [69]** | Malawi | retrospective cohort | district | 1986–1994 | 1,002 | < 30 to > 45 | TB/PTB/EPTB | all | unk/+/- | 147 (14.7%) ^4^ | 130 ** | tbx |
| **Gustafson 2007 [39]** | Guinea–Bissau | prospective cohort ^5^ | city | 1996–2001 | 292 | >= 15 | TB | all | +/- | 40 (13.7%) | 15 ** | 175.6 py tbx |
| **Hargreaves 2001 [31]** | Malawi | prospective cohort | city | 1997–1998 | 132 | unk | PTB | +/- | all | 36 (27.3%) | 6** | tbx |
| **Harries 1998 [33]** | Malawi | prospective cohort | district | 1995–1996 | 827 | 35 (s.d. 12) | TB/PTB/EPTB | +/- | +/- | 153 (18.5%) | 120 ** | tbx |
| **Harries 1999 [34]** | Malawi | prospective cohort | region | 1995–1996 | 170 | 35 (median) | TB | – | all | 64 (37.6%) | 4 | tbx |
| **Harries 2001 [81]** | Malawi | retrospective cohort | national | 1997 | 16004 | unk | PTB/EPTB | all/+/- | unk | 3720 (23.4%) | 3955 ** | tbx |
| **Horne 2010 [24]** | USA | prospective cohort | state | 1993-2005 | 3451 | 45 (s.d. 21) | TB | all | all | 88 (2.5% | 276 | median 5.9 yrs |
| **Hussey 1991 [86]** | South Africa | record review | city | 1985–1989 | 94 | 10.5 months (median) | ETB | n/a | unk | 13 (13.8%) | 0 | tbx |
| **Jochem 1997 [43]** | Nepal | prospective cohort | districts | 1990–1992 | 636 | unk | PTB | + | unk | 52 (8.2%) | 70 ** | tbx |
| **Kassim 1995 [38]** | Ivory Coast | prospective cohort | city | 1989–1992 | 835 | 35 (mean) | PTB | + | +/- | 61 (7.3%) | 144 ** | tbx |
| **Kelly 1999 [37]** | Malawi | prospective cohort | city | 1991–1993 | 121 | HIV +: 34.9  HIV-: 46  median | PTB | all | +/- | 29 (24.0%) | 4 ** | tbx |
| **Kim 2001 [60]** | Korea | retrospective cohort | region | 1988–1996 | 1,011 | 38.6 (13-79) | MDR PTB | all | unk | 3 (0.3%) | 442 ** | tbx |
| **Kolappan 2008 [70]** | India | retrospective cohort | district | 2000–2003 | 3,405 | 15 - >= 60 | TB | all | unk | 176 (5.2%) | 195 of 3,600 | tbx |
| **Lau 2005 [44]** | Hong Kong | prospective cohort | catchment area of 9 hospitals | 1993–2000 | 166 | 42.9 (3-100) | EPTB (TB meningitis) | n/a | 1.2% | 19 (11.5%)† | – | 3 years |
| **Leroy 1997 [53]** | France | retrospective cohort | region | 1988–1994 | 104 | 37 (s.d. 10.1) | TB | all | + | 11 (10.6%) † | 21 ** | 140 py |
| **Lillebaek 1999 [68]** | Denmark | retrospective cohort | national | 1992 | 210 | unk | PTB/EPTB | all | ≤ 5.2% | 7 (3.3%) † | – | tbx |
| **Low 2009 [79]** | Singapore | retrospective cohort | national | 2000-2006 | 7433 | < 45 to >= 65 | TB/EPT | all | all | 203 (2.7%)† | 0 | n.r |
| **Malkin 1997 [28]** | Burkina Faso | prospective cohort | city | 1988–1990 | 444 | HIV+: 33.4 (s.d. 10.6) HIV-: 39.2 (sd 16) | TB | all | +/- | 61 (13.7%) | n.r | tbx |
| **Mathew 2006 [59]** | Russia | retrospective cohort | oblast | 2002–2003 | 1,916 | 42 (18-88) median | TB | +/- | all | 138 (7.2%) † | – | tbx |
| **Mwaungulu 2004 [63]** | Malawi | retrospective cohort | district | 1999–2001 | 712 | 35 (s.d. 16) | TB/PTB/EPTB | all/+/- | +/- | 235 (33%) | 67 ** | tbx |
| **Najera–Ortiz 2008 [72]** | Mexico | retrospective cohort | region | 2002–2004 | 431 | 39.6 (s.d. 17.4) | PTB | all | all | 25 (5.8%) | 105 ** | tbx |
| **Noeske 2002 [52]** | Cameroon | prospective cohort | province | 1997-1998 | 560 | Males 33.8 (s.d. 11.1) females 31.8 (s.d. 12.3) | PTB | + | all | 47 (8.4%) | 56 ** | tbx |
| **Norval 1998 [64]** | Cambodia | retrospective cohort | national | 1994–1995 | 4,164 | unk | PTB | + | unk | 116 (2.8%) | 369 ** | tbx |
| **Nunn 1992 [50]** | Kenya | prospective cohort | city | 1989–1990 | 281 | <30 to > 40 | TB | all | +/- | 13 (4.6%)† | – | 6 months |
| **Okwera 1994 [88]** | Uganda | trial | city and surrounding area | 1990–1992 | 90 | 38 (s.d. 8) | PTB | + | + | 31 (34.4%) | 0 | tbx |
| **Pardesh 2009 [80]** | India | retrospective cohort | district | 2004 | 716 | < 20 to > 60 | TB | all | all | 41 (5.7%) | 0 | tbx |
| **Park 1996 [85]** | USA | record review | city | 1983–1993 | 152 | unk | MDR TB | all | +/- | 32 (21%)† | 0 | unk |
| **Perrriens 1995 [30]** | Zaire | prospective cohort | city and surrounding area | 1998–1992 | 523 | 31 (s.d. 7.3) | PTB | + | +/- | 20 (3.8%)† | 19** | tbx |
| **Perriens 1991 [87]** | Zaire | record review | city | 1987–1988 | 767 | HIV+: 31.8 (s.d.8.4); HIV -: 29.8 (s.d 10.7) | TB | all | +/- | 69 (9.0%) | 115 | tbx |
| **Sadacharam 2007 [46]** | India | prospective cohort | district | 2002–2003 | 1,171 | unk | PTB | + | unk | 58 (5.5%) | 25 | tbx |
| **Sakurai 1989 [47]** | Japan | prospective cohort | city | 1979–1983 | 1,083 | 30 - > 80 (70% >=50) | PTB | all | unk | 12 (1.1%) † | – | 3,845.75py |
| **Selby 1995 [67]** | United Kingdom | retrospective cohort | city and surrounding area | 1983–1992 | 730 | unk | TB | all | unk | 43 (5.9%) | n.r | unk |
| **Shen 2009 [76]** | China | retrospective cohort | city | 2000-2004 | 7,873 | 52 (11 to >-75) median | PTB/MDR TB | all/+/- | unk | 218 (2.8%) † | 126 | median 229 days |
| **Small 1991 [83]** | USA | record review | state | 1981–1988 | 125 | 36.4 median | TB | all | + | 8 (6.4%)† | n.r | tbx |
| **Thomas 2005 [71]** | India | retrospective cohort | district | 2000–2001 | 715 | unk | PTB | + | unk | 29 (4.1%) | 114 | tbx |
| **Van den Broek 1998 [27]** | Tanzania | prospective cohort | region | 1991 | 561 | unk | TB/PTB/EPTB | all/+/- | all/+/- | 56 (10.5%) | 70 ** | tbx |
| **Vijay 2004 [45]** | India | prospective cohort | city | 1999–2002 | 271 | 33 (13-82) | PTB | + | unk | 6 (2.2%) | 67 | tbx |
| **Vree 2007 [36]** | Vietnam | prospective cohort | national | 2002 | 1,881 | 15 to ≥ 55 | PTB | + | unk | 82 (4.4%) | n.r | tbx |
| **Walpola 2003 [56]** | Australia | retrospective cohort | state | 1989–1998 | 1,003 | 0 to ≥ 46 | TB | all | all | 87 (8.7%) † | 80 of 1,083 | tbx |
| **Wang 2002 [65]** | Taiwan | retrospective cohort | city | 1994–1999 | 39 | 40 (16 to 80) median | EPTB (TB meningitis) | n/a | unk | 2 (0.05%) | 0 | tbx |
| **Wang 2009 [77]** | Canada | retrospective cohort | national | 1997-2006 | 2,331 | - ( < 15 to ≥ 75) | TB | all | +/- | 146 (6.3%) | 50** | tbx |
| **Wilkinson 1996 [35]** | South Africa | retrospective cohort | city | 1993–1994 | 262 | HIV+: (23 to 38)  HIV-:(24 to 50) | TB | all | +/- | 27 (10.3%) | 28 | tbx |
| **Wilkinson 1998 [62]** | South Africa | retrospective cohort of children | district | 1991–1995 | 600 | < 16 | TB | all | all | 24(4%) | 0 | tbx |
| **Xie 1992 [84]** | Canada | Record review | national | 1980–1984 | 1,836 | unk | TB | unk | unk | 97 (5.3%) † | – | mean 568 days |
| **Zachariah 2007 [82]** | Malawi | retrospective cohort | district | 2004-2005 | 658 | 33 (1 to 74) median | TB | all | pos | 132 (20.0%) | 17** | tbx |
