# Supplementary material for: Assessing Tuberculosis Case Fatality Ratio: A Meta-Analysis
Source: PLoS One. 2011 Jun 27;6(6):e20755. doi: 10.1371/journal.pone.0020755 (PMC3124477; doi:10.1371/journal.pone.0020755)
Supplement: Table S2 — * MDR = multidrug resistant TB; PTB = pulmonary TB; † TB at least a major contributor to death in HIV– positive individuals; ‡ Of the 42 patients who died, tuberculosis was listed on the death certificate as the primary cause of death in 16 (38%), as a contributing cause of death in 15 (36%), TB was considered a major contributing cause of death for 4 deaths during the 6–month treatment period, 16 deaths were considered to be related to TB because they occurred before the response to anti TB therapy could be assessed; ** mortality during first 6 months of 12 months treatment duration; †† TB was recorded as immediate cause of death if death occurred within one month after initiating TB treatment and there was no other recorded causes. In patients with multiple cause of death the immediate cause was determined by review of the clinical data and the establishment of consensus. ‡‡ Autopsy results were available from 55 of 63 deaths. For 5 of 63 TB deaths HIV status was undocumented while 58 deaths were HIV-. These 63 TB deaths include 45 smear+ TB deaths. (DOC) [file pone.0020755.s002.doc]

**Table S2** Percentage (%) of deaths due to tuberculosis among treated tuberculosis patients that died during tuberculosis treatment.

| **Author, Location** | **Study period** | **Subgroup** | **% mortality**  **During TB treatment** | **Source** | **% mortality**  **due to TB**  **during TB**  **treatment** | **% of total death**  **due to TB**  **during TB treatment** |
| --- | --- | --- | --- | --- | --- | --- |
| *Small, USA [83]* | 1981–1988 | HIV positives | 40.8% (51/125) | review of available data **^†^** | 6.4% (8/125) | 15.7% (8/51) |
|  |  |  |  |  |  |  |
| *Perriens, Zaire [30]* | 1989–1991 | HIV positives | 13.1% (44/335) | *not specified* | 6.0% (20/335) | 45.5% (20/44) |
| *Boeree, Malawi [90]* | 1998–2001 | HIV positives | 14.7% (85/579) | clinical data, interview relatives | 3.8% (22/579) | 25.9% (22/85) |
| *Cain, Thailand [23]* | 2005-2007 | HIV positives | 16.7% (142/849) | study forms, death certificates, medical records, verbal autopsy reports | 4.4% (38/849) | 26.7% (38/142) |
| *Nunn, Kenya [50]* | 1989–1990 | HIV positives | 16.8% (18/107) **^**^** | review clinical data and consensus | 6.5% (7/107) **^††^** | 38.9% (7/18) |
| *Park, USA [85]* | 1983–1993 | HIV positives/MDR**^*^** | 61.5% (48/78) | medical records, death certificates, autopsy results | 28.2% (22/78) | 45.8% (22/48) |
|  |  |  |  |  |  |  |
| *Perriens, Zaire [30]* | 1989–1991 | HIV negatives | 0.0% (0/188) | *not specified* | 0.0% (0/188) | – |
| *Nunn, Kenya [50]* | 1989–1990 | HIV negatives | 4.6% (8/174) **^**^** | review clinical data and consensus | 3.4% (6/174) **^††^** | 75.0% (6/8) |
| *Park, USA [85]* | 1983–1993 | HIV negatives /MDR | 17.9% (7/39) | medical records, death certificates, autopsy results | 12.8% (5/39) | 71.4% (5/7) |
| *Alavi, Iran [75]* | 2002-2006 | overall | 3.2% (125/3960) | *medical records* | 2.3% (93/3960) | 74.4% (93/125) |
| *Borgdorff, the Netherlands [66]* | 1993–1995 | overall | 5.9% (258/4,340) | *not specified* | 0.8% (35/4,340) | 13.6% (35/258) |
| *Walpola, Australia, [56]* | 1989–1998 | overall | 12.7% (127/1,003) | review of available data | 8.7% (87/1,003) | 68.5% (87/127) |
| *Mathew, Russia [59]* | 2002–2003 | overall | 9.6% (183/1,916) | death certificates, autopsy reports, hospital medical charts | 7.2% (138/1,916) | 75.4% (138/183) |
|  | 2002-2003 | smear positives | 15.6% (143/919) | death certificates, autopsy reports, hospital medical charts | 11.9% (109/919) | 76.2% (109/143) |
|  | 2002-2003 | smear negatives | 4.0% (40/997) | death certificates, autopsy reports, hospital medical charts | 2.9%(29/997) | 72.5% (29/40) |
| *Lillebaek, Denmark [68]* | 1992 | Culture positive/ PTB**^*^** | 8.1% (17/210) | clinical records | 3.3% (7/210) | 41.2% (7/17) |
| *Fielder, USA [57]* | 1993–1998 | smear positives | 24.1% (42/174) | death certificates | 17.8% (31/174) ‡ | 73.8% (31/42) |
| *Shen, China [76]* | 2000-2004 | Culture positive/PTB | 5.9% (440/7,873) | death certificates | 2.8%(218/7,873) | 49.5%(218/440) |
