# Supplementary material for: Assessing Tuberculosis Case Fatality Ratio: A Meta-Analysis
Source: PLoS One. 2011 Jun 27;6(6):e20755. doi: 10.1371/journal.pone.0020755 (PMC3124477; doi:10.1371/journal.pone.0020755)
Supplement: Table S3 — * Categorization subgroups: Human Immunodeficiency Virus positive (HIV pos.) or negative (HIV neg.); Smear positive (Sm pos.) or negative (Sm neg.); † Unless specified otherwise follow up time was calculated from the initiation of TB treatment; § Unless specified otherwise, mortality is assessed as percentage of original TB cohort initiating TB treatment without adjustment for percentage of patients lost to follow up; 1a 999,7 person years (p.yrs) follow up time for HIV pos. and 560 p.yrs follow up time for HIV neg., 1b at 12 months 11.7% loss to follow up (ltfu); 2a 113,4 p.yrs follow up time for HIV pos. and 123,1 p.yrs follow up time for HIV neg., 2b at 12 months 6% ltfu; 3a 19% (33/174) of HIV pos and 17% (11/65) of HIV neg. was ltfu at end of treatment, 3b 25% (44/174) of HIV pos. and 26% (17/65) of HIV neg. was lftu at the end of follow up period; 4 HIV status was available for 96% (793/827) registered TB patients, 11.9% (99/827) registered TB patients was ltfu on 32 months and 18% (152/827) registered TB patients was ltfu at 7 years; 5a at end of 6 months treatment regimen 6.1% (22/359) defaulted, was ltfu or transferred out, and 12% (42/359) was ltfu at end of 2 years follow up, 5b at end of 12 months treatment regimen 21% (63/304) defaulted, was ltfu or transferred out, and 20% (61/304) was ltfu at end of 2 years follow up; 6 at end of treatment 5% (195 of 3,600 patients starting TB treatment) could not be followed up because they migrated, transferred out or could not be traced; these are excluded from the denominator in all analyses; 7 at the end of treatment 13% (68/512) was loss to follow up and these were excluded from the denominator in all analyses; 8a at end of treatment records were unavailable for 12% (20/170) HIV pos. and 16% (96/597) HIV neg., 8b 39% (67/170) of initial cohort was followed up in HIV pos. and 43% (258/597) in HIV neg.; 9 at end of treatment 2% (25/1,171) was ltfu, 46 could not be traced during follow up consequently a total of 6% [file pone.0020755.s003.doc]

## Table S3 Proportion of deaths during follow up after initiating TB treatment

| **Author, Location** | **Subgroup ^*^** | **Data source or**  **follow up** | **Follow up,**  **after initiating**  **TB treatment ^†^** | **% mortality (n^1^/N)**  ***during* TB treatment ^§^** | **% mortality**  ***after* TB**  **treatment (n^2^/N)** | **% total**  **mortality**  ***end* follow up**  **(n^1+2^/N)** | **% of total deaths dying *after* TB treatment**  **(n^2^/ n^1+2^)** |
| --- | --- | --- | --- | --- | --- | --- | --- |
| Ciglinecki, Zambia, [89]  Ciglinecki, Zambia, [89] | HIV pos.  HIV neg. | Clinic visits  Clinic visits | 1.6 years ^1a^  1.6 years ^1a^ | 14% (98/676)  1% (2/320) | 12% (84/676)  3% (8/320) | 27% (182/676) ^1b^  3% (10/320) ^1b^ | 46% (84/182)  80% (8/10 ) |
| Ciglinecki, Malawi, [89]  Ciglinecki, Malawi, [89] | HIV pos.  HIV neg. | Clinic visit  Clinic visit | 1.6 years ^2a^  1.6 years ^2a^ | 21% (18/84)  2% (1/65) | 19% (16/84)  5% (3/65) | 40% (34/84) ^2b^  6% (4/65) ^2b^ | 47% (16/34)  75% (3/4) |
| De Riemer, Mexico [26] | Sm pos. | Annual follow up | Not reported | 5% (22/436) | 4% (17/436) | 9% (39/436) | 77% (17/22) |
| Elliot, Zambia [29] | HIV pos. | Clinic and home visits | < 24 months | 29% (51/174) ^3a^ | 13% (23/174) | 43% (74/174) ^3b^ | 69% (51/74) |
| Elliot, Zambia [29] | HIV neg. |  | < 24 months | 8% (5/65) ^3a^ | 3% (2/65) | 11% (7/65) ^3b^ | 71% (5/7) |
| Garin, Central African Republic [48] | HIV pos.  HIV neg. | Review National TB Register (NTR) | 24 months  24 months | 39% (54/139)  8% (7/85) | 19% (27/139)  12% (10/85) | 58% (81/139)  20% (17/85) | 33% (27/81)  59% (10/17) |
| Harries, Malawi [33,104,105] | HIV pos. | Home visit | 2.7 years [27]  7 years [87] | 34% (206/612) [88] | 13% (79/612) ^4^  36% (223/612) ^4^ | 47% (285/612)  70% (429/612) | 28% (79/285)  52% (223/429) |
|  | HIV neg. | Home visit | 2.7 years [27]  7 years [87] | 17% (31/181) | 9% (17/181)  31% (57/181) | 27% (48/181)  49% (88/181) | 35% (17/48)  65% (57/88) |
| Jochem, Nepal [43] | Sm pos.  Sm pos. | Clinic and home visits | 2 years  2 years | 5% (18/359) ^5a^  11% (34/304) ^5b^ | 3% (9/359)  5% (16/304) | 7.5% (27/359) ^5a^  13% (40/304) ^5b^ | 33% (9/27)  4  0% (16/40) |
| Kassim, Ivory Coast, [38] | HIV pos.  HIV neg. | Clinic and home  visits | 2 years  2 years | 10% (57/553)  1% (4/282) | 12% (64/553)  2% (6/282) | 22% (121/553)  4% (10/282) | 53% (64/121)  60% (6/10) |
| Kolappan, India [70] | *overall* | Home visit | 3.3 years | 5% (176/3,405) ^6^ | 15% (519/3,405) | 20% (695/3,405) | 75% (519/695) |
| Malkin, Burkina Faso [28] | HIV pos.  HIV neg. | Study follow up  and home visit | 1.5 years  1.5 years | 27% ^7^ (26/96)  10% ^7^ (35/348) | 7% (7/96)  3% (10/348) | 34% (33/96)  13% (45/348) | 21% (7/33)  22% (10/45) |
| Perriens, Zaire [87] | HIV pos. | Clinic | mean 0.85 year | 28% (47/170) ^8a^ | 9% (15/170) | 36% (62/170) ^8b^ | 24% (15/62) |
|  | HIV neg. |  | mean 0.90 year | 4% (22/597) ^8a^ | 1% (5/597) | 5% (27/597) ^8b^ | 19% (5/27) |
| Sadacharam, India, [46] | Sm pos. | Home visit | 2–3 years | 5% (58/1,171) ^9^ | 13% (148/1,171) ^2^ | 18% (206/1,171) | 72% (148/206) |
| Small, USA [83] | HIV pos. | Record review by panel | 9.6 months  after treatment completion | 41% (51/125) ^10^ | 36% (45/125) | 77% (96/125) | 47% (45/96) |
| Van den Broek, Tanzania [27] | HIV pos.  HIV neg. | Home visit, relatives and neighbors | 3 years  after treatment completion | 16% (24/146) ^11^  8% (32/415) ^11^ | 18% (27/146)  6% (24/415) | 35% (51/146)  13% (56/415) | 53% (27/51)  43% (24/56) |
| Vijay, India [45] | Sm pos. | Home visit | 2.5 years | 2% (6/271) ^12a^ | 13% (35/271) ^12b^ | 15% (41/271) | 85% (35/41) |
